# Optimal Design of Carbon-Based Polymer Nanocomposites Preparation Based on Response Surface Methodology

**DOI:** 10.3390/polym15061494

**Published:** 2023-03-17

**Authors:** Shaoqiu Yan, Ying Tang, Gangping Bi, Bowen Xiao, Guotian He, Yuanchang Lin

**Affiliations:** 1 Chongqing Institute of Green Intelligent Technology, Chinese Academy of Sciences, Chongqing 400714, China; 2 College of Mechanical Engineering, Chongqing University of Technology, Chongqing 400054, China; 3 Chongqing Key Laboratory of Artificial Intelligence and Service Robot Control Technology, Chongqing Institute of Green Intelligent Technology, Chinese Academy of Sciences, Chongqing 400714, China; 4 College of Artificial Intelligence, Chongqing School, University of Chinese Academy of Sciences, Chongqing 400020, China

**Keywords:** response surface, central composite design, CNT/GN, sensitivity, compressive modulus

## Abstract

Response surface methodology (RSM) and central composite design (CCD) were used to improve the preparation of carbon nanotube and graphene (CNT-GN)-sensing unit composite materials in this study. Four independent variable factors (CNT content, GN content, mixing time, and curing temperature) were controlled at five levels, and 30 samples were generated using the multivariate control analysis technique. On the basis of the experimental design, semi-empirical equations were developed and utilized to predict the sensitivity and compression modulus of the generated samples. The results reveal a strong correlation between the experimental and expected values of sensitivity and the compression modulus for the CNT-GN/RTV (room-temperature-vulcanized silicone rubber) polymer nanocomposites fabricated using different design strategies. The correlation coefficients for the sensitivity and compression modulus are $R^{2\;}=0.9634$ and $R^{2}=0.9115,$ respectively. The ideal preparation parameters of the composite in the experimental range include a CNT content of 1.1 g, a GN content of 1.0 g, a mixing time of 15 min, and a curing temperature of 68.6 °C, according to theoretical predictions and experimental findings. At 0~30 kPa, the CNT-GN/RTV-sensing unit composite materials may reach a sensitivity of 0.385 kPa^−1^ and a compressive modulus of 601.567 kPa. This provides a new idea for the preparation of flexible sensor cells and reduces the time and economic cost of experiments.

## 1. Introduction

Polymer nanocomposite is a composite material with polymer as the continuous phase of the matrix and nanoscale fillers as the dispersed phase. The fillers are homogeneously dispersed in the polymer matrix, resulting in synergistic effects depending on the characteristics of different polymer matrix and filler materials, thus giving the material distinctive mechanical [1], electrical [2], optical [3], and biocompatible [4] properties. Polymer nanocomposites are now widely used in the field of stress/strain sensing [5]. Among them, the sensitivity and the compression modulus are two key metrics that affect the comprehensive performance of sensors, which represent the tactile and mechanical properties of sensors, respectively. However, the fabrication of sensing materials with excellent sensitivity and compression modulus performance is severely constrained by the complexity of sensing systems. Therefore, the development of polymer nanocomposite-sensitive units with high performance and stability for sensors has attracted extensive attention from researchers worldwide.

Owing to their superior mechanical and electrical conductivity, carbon-based nanomaterials are frequently employed in the construction of high-performance polymer composites. Carbon nanotubes and graphene have been widely explored and used due to their distinctive structures, electrical characteristics, mechanical capabilities, and chemical stability [6,7], making them significant nano-filling materials for polymers. As a one-dimensional conductive nanomaterial, carbon nanotubes have a small percolation threshold and excellent electrical conductivity due to their nanoscale and high aspect ratio. Chen [8] used AgNWs as the conductive layer PDMS and as a flexible substrate and used the polyol reduction method to prepare a flexible strain sensor with a strain sensitivity of 2.875 and a good linear relationship between resistance and strain. Nayak [9] modified multi-walled carbon nanotubes (MWCNTs) by employing polycarbosilane-derived silicon carbide to improve the dispersion of MWCNTs in the polymer matrix, thereby improving the thermal and mechanical properties of the nanocomposites. Graphene, consisting of a single layer of sp2-hybridized carbon atoms [10], is a conductive, two-dimensional material that has been highly used in recent years; with high carrier mobility and a large specific surface area, it is an ideal carbon-based conductive filler. Zheng [11] proposes a flexible graphene sensor based on a bionic spider web structure using laser-induced graphene as a spider web structure and encapsulating it on a PDMS substrate, which exhibits a wider operating range. Tang et al. [12] added graphene to overcome the low conductivity and toughness problems of wearable sensors, thus making sensors with a high conductivity level of 10^6^ s/m and exhibiting very good mechanical toughness, reversible deformability, and structural stability. Graphene was modified and graphene oxide was obtained to combine with some functional groups of the polymer to make it structurally stable, and the resulting sensor possessed 75% high compressibility [13]. The π−π interactions and van der Waals forces may cause an agglomeration of nanofiller materials due to weak dispersion in the polymer matrix, which reduces the sensing and mechanical properties despite the preparation of a variety of flexible sensors doped with graphene and carbon nanotubes using different methods. Thus, a reasonable fit ratio of graphene and carbon nanotubes in the polymer is essential. However, these preparation methods typically change only one study factor while leaving the others unchanged, ignoring the interactions and effects of each factor to such an extent that the optimal conditions for preparing high-performance flexible sensors cannot be achieved. Therefore, it is essential to create an efficient and effective technique for the production of high-performance, graphene-doped-carbon, nano-flexible sensors.

Response surface methodology (RSM), which employs a rational experimental design method and obtains related data through experiments, then employs a multiple quadratic regression equation to fit the functional relationship between the factors and response values and then solves multivariate problems by analyzing the regression equation to determine the optimal process parameters [14]. Several researchers have optimized the properties of polymeric nanomaterials based on response surface methodology techniques. For example, Moayad used the central composite design method (CCD) in RSM to evaluate the effect of different temperatures and magnetic filler content on the thermal transfer properties of NiZn/TPNR nanocomposites and to predict and optimize the thermal conductivity values of the composite [15]. Two crucial components of RSM are the design of experiments (DOE) and regression analysis. The DOE is a systematic approach for establishing the link between input factors and output response variables and for generating design samples, while regression analysis evaluates the degree of variance in the response variables as a result of the effect of the independent variables [16]. The quantitative analysis of the process parameters through RSM is used to identify the primary variables and control the factors related to them in order to obtain the best experimental results with the fewest number of tests, thereby improving the performance of the samples and optimizing the preparation process. Hamed et al. [17] used the Box–Behnken experimental design method in RSM to investigate the effects of mixing time, mixing speed, and CNT concentration on the electrical, mechanical, and strain-sensing properties of epoxy nanocomposites, and developed two regression equations to predict their electrical conductivity and tensile strength. The results show that the nanocomposites prepared at high mixing speeds and under a long mixing time have better sensing properties. Thus far, however, there has been little research on the optimization of various aspects influencing the fabrication of graphene-carbon nanotube-flexible sensors utilizing the RSM approach.

Using the RSM approach and CCD, this study studied the impacts of four independent variable parameters, including CNTs dosage, GN dosage, curing temperature, and mixing time, on the sensitivity coefficient and compression modulus. The effective parameters were adjusted using the statistical program Design Expert, and optimal RSM models of sensitivity coefficient and compression modulus were developed. Finally, the optimal range of preparation settings for flexible graphene–carbon nanotube sensors’ sensing performance and mechanical qualities was identified.

## 2. Materials and Methods

### 2.1. Design of the Central Combination of CNT-GN/RTV Polymer Nanocomposites

**Figure 1:**
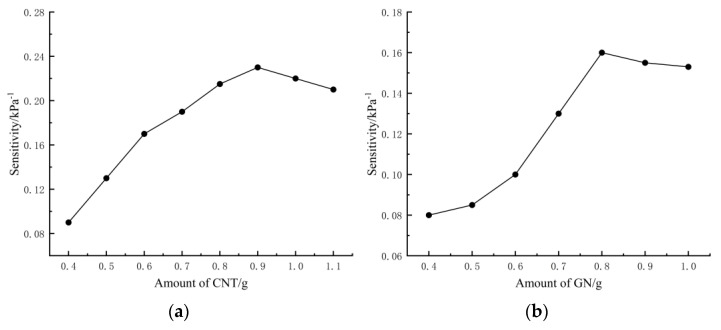

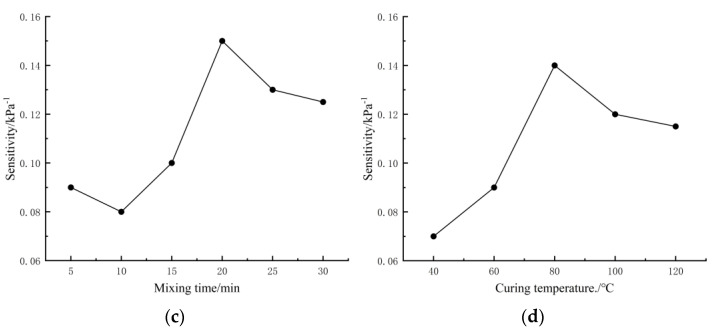
Effect of individual factors on sensor sensitivity. (**a**–**d**) are the effects of CNT, GN, mixing time, and the curing temperature on sensitivity, respectively.

The protocol for testing CNT-GN/RTV polymer nanocomposites was developed using a central composite design (CCD). The effects of four factors—CNT content, GN content, mixing time, and curing temperature—on the flexible sensors were investigated on a single-factor basis, and the sensitivity and compression modulus were chosen as the response values, where sensitivity was used as the main response value for the single-factor experimental design, as shown in Figure 1. The four independent variables were converted to dimensionless variables (A, B, C, and D) with coded values of −2, −1, 0, +1, and +2. The coding levels are shown in Table 1. Factor A is 0.7, 0.8, 0.9, 1.0, and 1.1 g; factor B is 0.6, 0.7, 0.8, 0.9, and 1.0 g; factor C is 10, 15, 20, 25, and 30 min; and factor D is 40, 60, 80, 100, and 120 °C.

As indicated in Table 2, the experimental design was produced using Design Expert (trial version 11, Stat-Ease, Inc., Minneapolis, MN, USA) software and the center combination design approach. It is mostly comprised of $2^{m}$ factorial operations, 2m axial point operations, and *C* center point operations, and the experiment number of repetitions for each process variable may be computed using Equation (1).

(1) $$N=2^{m}+2m+C=2^{4}+\left(2\times 4\right)+6=30$$

where N is the required number of experiments and m is the number of impact variables.

CCD consists of three processes: performing design experiments, computing model coefficients, and forecasting the model’s accuracy and reliability. Therefore, an empirical model is required to compute the response values and establish a functional link with the input processes’ components. This process is represented by a quadratic regression model [18,19]. Equation (2) demonstrates the functional connection between the response values and the influencing variables.

(2) $$Y=b_{0}+\overset{k}{\underset{i=1}{\sum }}b_{i}X_{i}+\overset{k}{\underset{i=1}{\sum }}b_{ii}X_{i}^{2}+\overset{k-1}{\underset{i=1}{\sum }}\overset{k}{\underset{j=2}{\sum }}b_{ij}X_{i}X_{j}$$

where Y*,* $b_{0},b_{i},b_{ii}$*,* and $b_{ij}$ represent the predicted response values, constant coefficients, linear coefficients, quadratic equations, and interaction coefficients, respectively. $X_{i},X_{i}^{2}$, and $X_{i}X_{j}$ represent the linear, quadratic, and interaction effects of the independent variables (CNT content, GN content, mixing time, and curing temperature), respectively.

The reliability of the polynomial model equations can be evaluated by the values of $R^{2},\;\;R_{Adj}^{2}$, and $R_{Pred}^{2}$. When the correlation coefficient ($R^{2}$) is comparatively high, it is proven that the experimental data fit well with the model [20]. Meanwhile, the interaction between the four independent variable factors can be calculated using a one-way analysis of variance (ANOVA), and when the factor P<0.05, it means that the factor is a significant parameter and has a significant effect on the model.

### 2.2. Preparation of Samples

**Figure 2:**
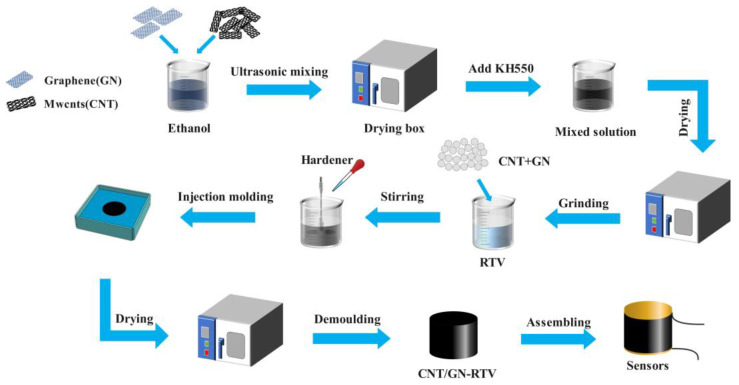
The example flowchart for the manufacturing of CNT/GN-RTV polymers.

Mechanical blending involves the addition of two or more types of nanoparticles into a polymer matrix [21]. CNTs and GN nanofillers were added to the RTV in accordance with Table 1’s mass ratio. The purity of the CNTs was larger than 95 wt%, their length was larger than 3 µm, and their outer diameter was 8–15 nm. GN with 5–10 layers and an average diameter of 50 µm were provided by Suzhou Tan-Feng Graphene Technology Co., Ltd., Suzhou, China. According to Figure 2, before preparation, CNT and GN were added to an aqueous ethanol solution (Guangdong Wengjiang Chemical Reagent, Shaoguan, China) in a volume ratio of 3:1 and ultrasonically dispersed for 10 min, followed by the high-temperature drying of the prepared conductive particles. Then, a certain proportion of KH550 (Jiangsu Runfeng Synthesis Technology Co., Nantong, China) was added to the resulting mixed solution, which was placed in a 100 °C high-temperature drying oven (Shanghai Heheng Instrument Equipment Co., Shanghai, China) for drying; after drying, the modified conductive filler was finely ground with a mortar. The RTV matrix (Shenzhen Red Leaf Jie Technology Co., Shenzhen, China) was then mixed using a stirrer (JJ-1B, Youlian Instruments, Changzhou, China) after the modified conductor filler was introduced. RTV is a non-toxic, non-polluting silicone material that can be cured at room temperature without heating or pressure, and is an elastic colloid with good wear resistance and chemical stability after curing, so it was chosen as the base material for preparing sensors. The curing ingredient was added according to a mixing ratio of 10:1 and mixed for one minute. The mixture was then injected into the mold and placed in a vacuum chamber (RS-1, Dawei Electromechanical, Weihai, China) for 20 min of air venting. After air venting, the mold was placed in a high-temperature drying oven for three hours to dry and was then returned to room temperature for natural curing.

### 2.3. Sensitivity of CNT-GN/RTV Polymer Nanocomposites

Sensitivity (GF) is a key performance metric for capacitive sensors and is often represented as the ratio of capacitance change to stress change [22]. The capacitance data was obtained by forming a test circuit with a microcomputer-controlled electronic universal testing machine (FBS200N, Forbes Testing Equipment Co., Ltd., Xiamen, China) and an LCR digital bridge (UC2836B, U-Tech Electronics Technology Co., Ltd., Suzhou, China), placing the sample in the testing machine, applying 10 N pressure to the sample to cause it to deform, and then monitoring and collecting the sample capacitance data with the LCR digibridge (20 Hz). The sensitivity was calculated according to Equation (3):

(3) $$GF=\Delta C/\Delta PC_{0}$$

where GF represents sensitivity (kPa^−1^); $C_{0}$ is the initial capacitance without applied pressure (pF); $\Delta C=C-C_{0}$ denotes the amount of capacitance change, where C is the capacitance value under compression; and ΔP represents the applied pressure (kPa).

### 2.4. Compressive Modulus of CNT-GN/RTV Polymer Nanocomposites

The ultimate distance between the sensor poles is defined by the sensor’s capacity for compressive deformation, and the elastic modulus indicates the material’s resistance to elastic deformation [23]. The lower the material’s elastic modulus, the more easily it is deformed by force and the more sensitive it is to pressure. Therefore, the fabrication of nanocomposites with a low elastic modulus may efficiently increase the sensor’s sensitivity.

The prepared sample (r = 10 mm, h = 10 mm) was placed between two compression plates of a universal testing machine, and a force of 10 N was applied at a constant rate of 1 mm/min along both end faces of the specimen to produce compressive deformation. The sample was shortened in the axial direction and elongated in the radial direction. The testing equipment recorded the applied force and calculated the compressive modulus based on Equation (4):

(4) E=σ/ε

where E indicates the compressive modulus, σ is the compressive stress (kPa), and ε represents the compressive strain of the sample.

## 3. Results and Discussion

### 3.1. ANOVA and Model Fitting for CNT/GN-RTV Nanocomposites’ Sensitivity

Table 3 displays the ANOVA table for the sensitivity response surface model. The model’s *p*-value was less than 0.00001 and the *F*-value was 28.18, suggesting that it was very significant. Since the *p*-value for the lack-of-fit term was greater than 0.05 and not statistically significant, there was no lack-of-fit factor. When the *p*-value of the primary influence factor is less than 0.05, the component has a significant effect on the model. A, B, C, D, AB, AC, AD, BC, BD, $A^{2}$, and $B^{2}$ were important model terms in this scenario, indicating that they have a very significant impact on the sensitivity of CNT/GN-RTV nanocomposites. An interaction exists between the components, as shown by the large differences in the partial regression coefficients of AB, AC, AD, BC, BD, $A^{2}$, and $B^{2}$, and the model’s response values are not just linear connections [24]. The coefficient of variation (CV) for the model was 14.95%; a low CV number indicates a high degree of confidence in the model’s ability to properly reflect the actual experimental data. The model had an $R^{2}=0.9634$ and an $R_{Adj}^{2}=0.9292$, showing that the model was well-fitting and that the regression equation could be used to assess the experimental results. According to the aforementioned results, the composite’s sensitivity was impacted by the CNT content, the GN content, the mixing period, and the curing temperature. When coupled with the *F*-value, the relationship between the experimental components of the model could be deduced: CNT content preceded GN content, curing temperature, and mixing time.

On the basis of the central combination idea, a response surface analysis was developed, and Table 2’s data were fitted using multiple regressions. The sensitivity of CNT/GN-RTV nanocomposites was chosen as the response variable. The input variables were CNT content, GN content, mixing time, and curing temperature, which formed a functional relationship between the response variable and the input variables. It may be represented as a coding factor, as shown in the following equation:

(5) $$\begin{array}{cc} Sensitivity= & 0.142+0.090A+0.180B-0.021C+0.017AB-0.015AC+0.015A-0.019BC-0.029BD\\ & -0.003CD+0.033A^{2}+0.015B^{2}+0.008C^{2}-0.008D^{2} \end{array}$$

where sensitivity is the response variable of the composite, A and B are the corresponding CNT and GN weights, C is the mixing time, and D is the curing temperature.

**Figure 3:**
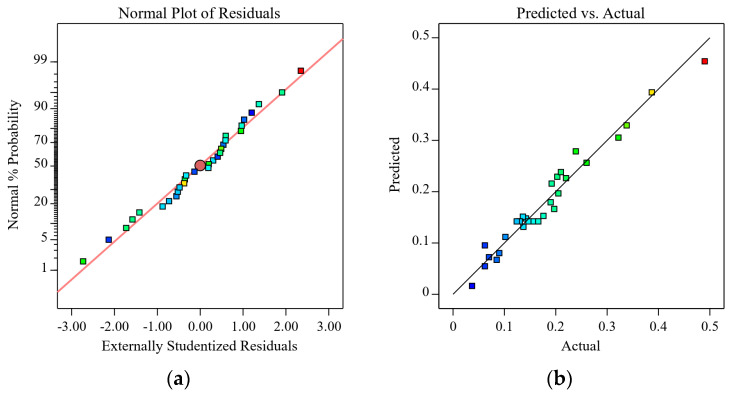
Normal probability plot of the residuals of the sensitivity of CNT/GN-RTV composites (**a**); (**b**) predicted versus actual values.

The normal probability plot of the residuals [25] represents the difference between the actual and predicted values (fitted values), and the data produced by the residuals is used to assess the model’s validity and the data precision. As observed in Figure 3a, the normal probability of the residuals was more equally distributed along a straight line, indicating that the errors were normally distributed and that the model and experimental data were consistent. As seen in Figure 3b, the actual and predicted sensitivity values were also more equally distributed along the diagonal line, further confirming the high and well-adapted fit of the regression equation and the high dependability of the generated model.

**Figure 4:**
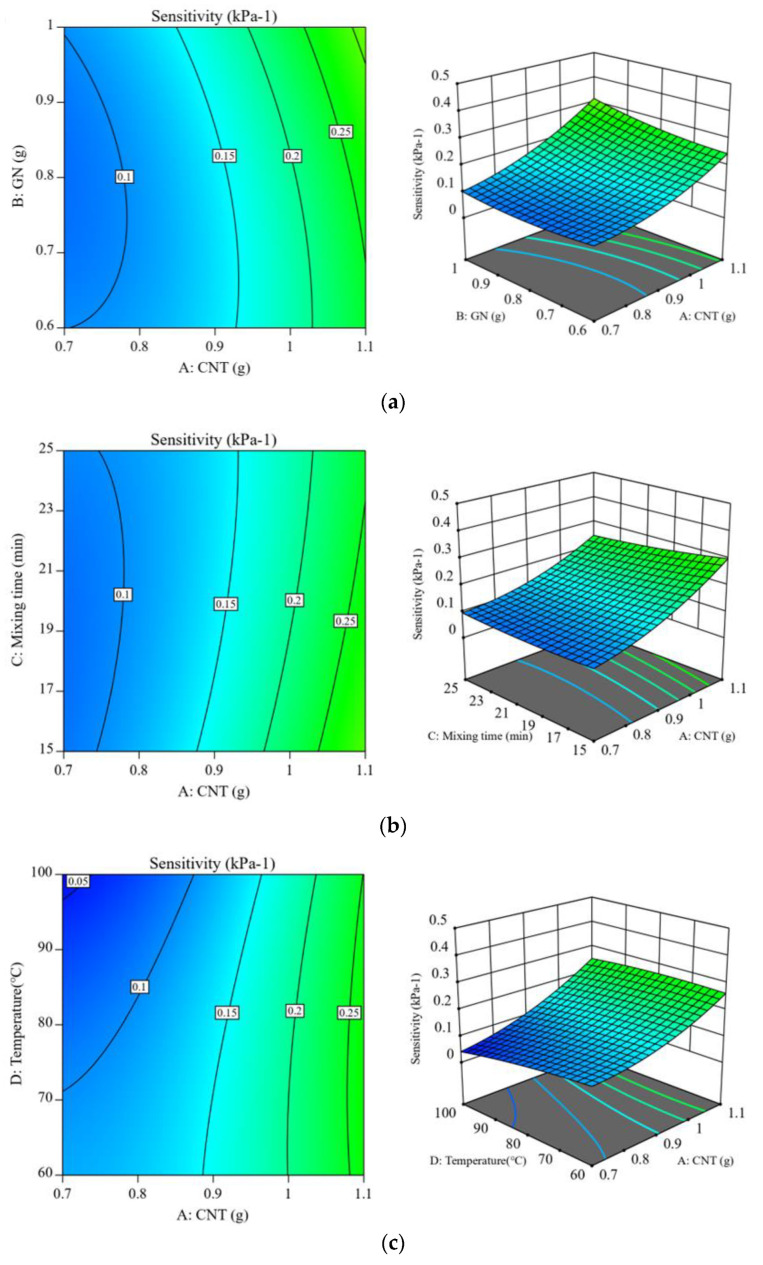

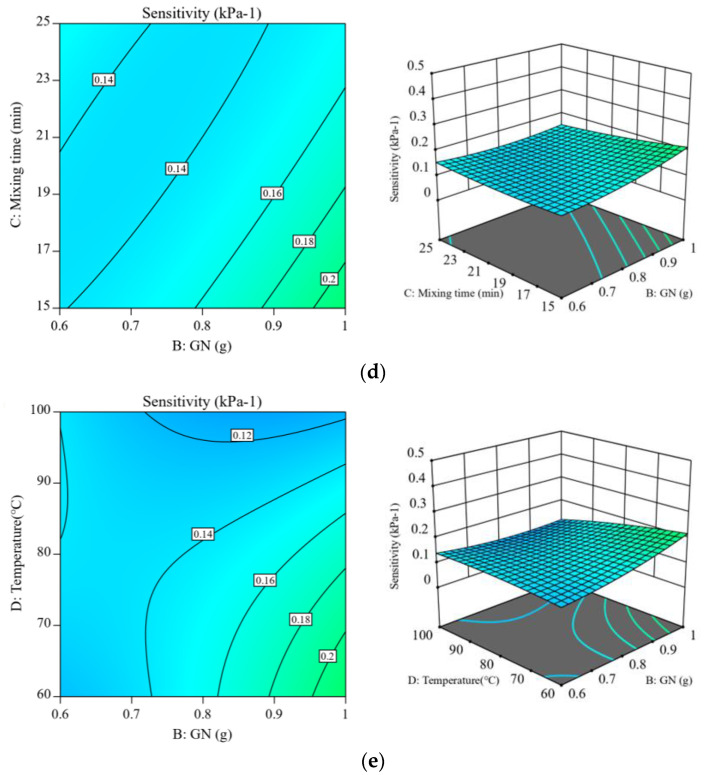
Studies of the relationships between (**a**) CNT and GN content, (**b**) CNT content and mixing time, (**c**) CNT content and curing temperature, (**d**) GN content and mixing time, and (**e**) GN content and curing temperature.

In line with the suggested model, three-dimensional response surfaces and contour plots were generated to analyze the interactions between the effects of CNT content, GN content, mixing time, and curing temperature on sensitivity. As demonstrated in Figure 4a, the amounts of CNT and GN had a substantial effect on the enhancement of sensitivity. This may be a result of the large specific surface areas of CNT and GN, which enable each CNT molecule to adsorb two GN molecules to form a three-dimensional mesh structure [26], thereby increasing the contact area with the RTV substrate. At the same time, the synergistic effect of the two increases filler dispersion and prevents conductivity loss from agglomeration. Figure 4b,d demonstrates that the improvement in sensitivity with increased mixing time at a given CNT or GN concentration was not significant; rather, an overly long mixing time may result in a decrease in sensitivity. This may be due to the fact that prolonged shear mixing [27] impedes the formation of the “network” and destroys the “bridge” between CNT and GN. For the improvement of sensitivity, a suitable mixing period is essential. Figure 4c,e demonstrates that the softening of the substrate during the high-temperature curing process led to a decrease in the distance between CNT and GN [28], which promotes the synergistic action of the mixed fillers. The nanofillers are, likewise, more readily aligned in the loading application-favorable orientation, but the reduction in interfacial quality, caused by the differing thermal expansion coefficients of the two fillers, reduces the sensitivity.

### 3.2. ANOVA and Model Fitting for the Compressive Modulus of CNT/GN-RTV Nanocomposites

The response surface regression model of the compressed modulus could be put through an ANOVA to increase the model’s credibility. The results of the response surface ANOVA of the compressed modulus are displayed in Table 4, where it can be seen that the model’s $R^{2}=0.9115$ and its *p*-value was less than 0.0001. This suggests that the model achieved a highly significant link and also demonstrates the validity of both the established model and the experimental approach. The residuals were generated by random errors and the lack-of-fit term (*p*-value = 0.8497) was not significant, which further suggests that the unknown factors had a minimal impact on the experiment. The misfit term’s F-value=0.4785, at the same time, and a lower F-value implies a better fit for the model.

The bigger the F-value, which denotes a more significant influence of the factor on the compression modulus, the smaller the value of the *p*-value corresponding to the factor in the response surface model. According to the model’s *p*-values, the values of C, D, AB, AC, BD, and $D^{2}$ were all less than 0.05, which denotes a more substantial impact on the response value. Additionally, it is evident from the *p*-value that the interaction of AB had a very significant impact on the response surface. AC and BD also had a more significant impact than AD, BC, and CD, which did not have a significant impact. We may infer the following connection between the four parameters in the model from the findings above: curing temperature > mixing time > GN content > CNT content. The four factors in the model each had a distinct degree of effect on the compression model.

In order to create a functional relationship between the response variable and the input variables, multiple regression was fitted to the data in Table 2 using the compressive modulus of the CNT/GN-RTV nanocomposite as the response variable and the CNT content, GN content, mixing time, and curing temperature as the input variables. It may be written as a coding factor, as seen by the equation below.

(6) $$\begin{array}{cc} \mathrm{Compression} & \mathrm{modulus}\\ & =595.78+7.26A+3.22B+23.18C-89.7D-10.11CD+5.6A^{2}+46.31AB+28.77AC\\ & +15.57AD+1.29BC+29.94BD-14.29B^{2}+8.35C^{2}-51.93D^{2} \end{array}$$

where C is the mixing time; D is the curing temperature; A and B are the weights of CNT and GN, respectively; and the compressive modulus is the composite’s response variable.

**Figure 5:**
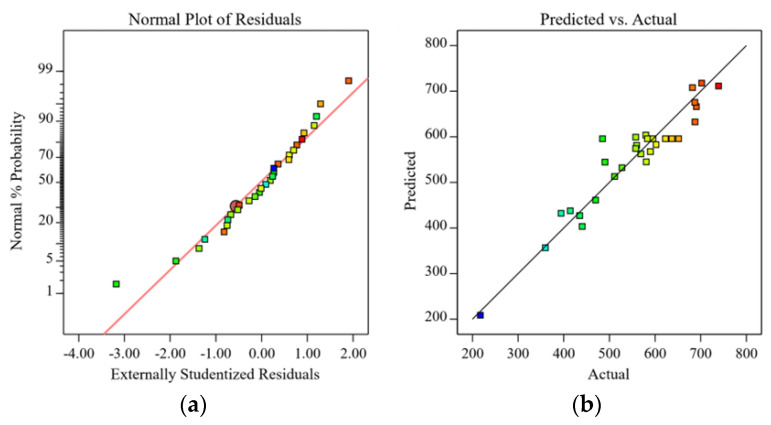
Normal probability plot of the residuals of the compression modulus of CNT/GN-RTV composites (**a**); (**b**) predicted versus actual values.

Figure 5a,b illustrates the normal probability plots of the residuals and the correlation between the actual and anticipated values. By using the compressive modulus data to examine the model, the normal probability plots of the residuals offer a visual evaluation of the model’s dependability. Additionally, the highly fitted values of $R^{2}$ (0.9115) and $R_{Adj}^{2}$ (0.8289) for the actual and projected values further support this conclusion.

**Figure 6:**
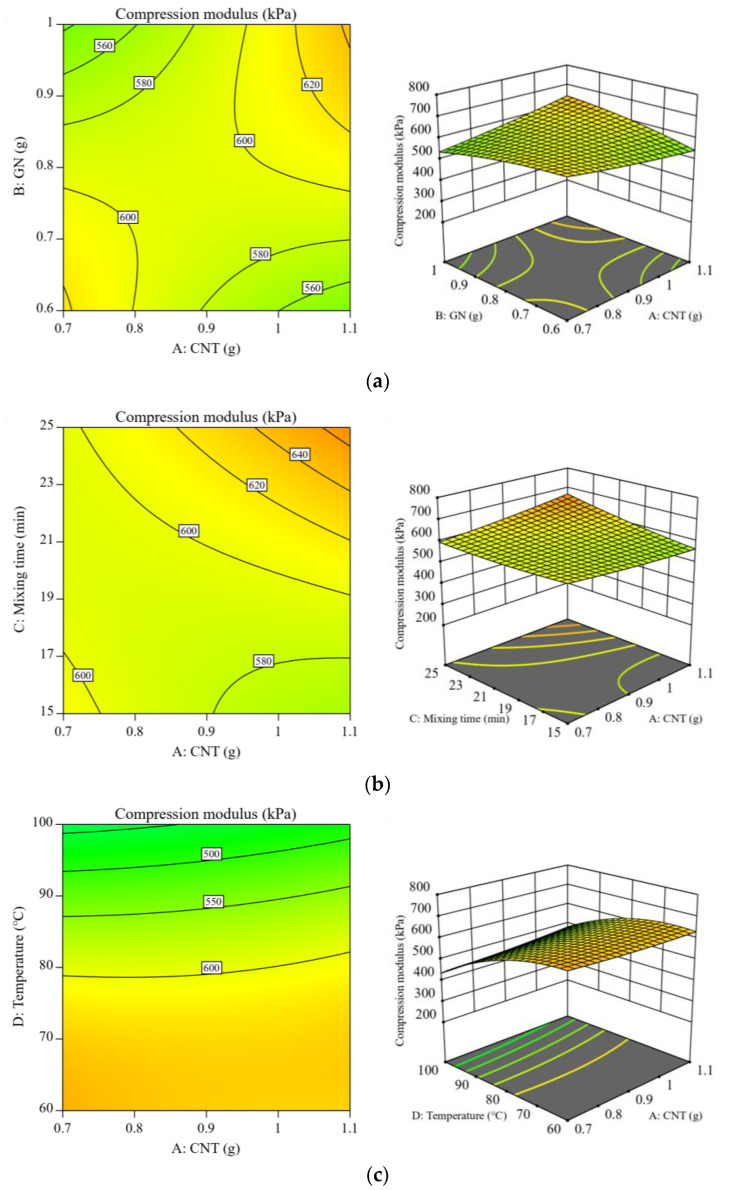
Studies of the relationship between the compressive modulus contour map and the three-dimensional response surface of CNT/GN-RTV composites with (**a**) CNT and GN content, (**b**) CNT content and mixing time, and (**c**) CNT content and curing temperature.

On the basis of the created model, three-dimensional response surfaces and contour plots were drawn to study the interaction of CNT content, GN content, mixing time, and curing temperature on the compression modulus. The contents of CNT and GN had a significant effect on the enhancement of the compression modulus, as shown in Figure 6a. This is conceivable, given that both GN and CNT nano-fillers have increased dimensionality and a larger surface area in contact with the polymer matrix, which increases the stress distribution and considerably enhances the interfacial impact [29]. Consequently, the compressive modulus of the composite increases with increasing CNT and GN content; nevertheless, the interactions and van der Waals forces in the nanostructure of the reinforcing materials utilized may promote polymer agglomeration, making filler quantity selection crucial. As demonstrated in Figure 6b, when the CNT content was held constant, the composite’s compressive modulus rose with increased mixing time. Agglomeration or an uneven distribution of the filler in the matrix might be a fault, but as mixing time increases, agglomeration decreases, hence decreasing the void ratio between the filler and the matrix. According to Figure 6c, the maximum compression modulus of the composite at 60 °C was about 620 kPa, and the modulus decreased continuously as the temperature increased. In the higher temperature curing environment, the RTV stock solution had a quicker molecular movement rate and a weaker bonding force with the curing agent, while the PDMS stock solution had a slower molecular movement rate and a stronger bonding force with the curing agent. The curing temperature had a significant effect on the mechanical properties of the composite matrix due to the entropic effect, which also facilitated a more complete silicon-hydrogen addition reaction and improved the crosslinking network in the RTV matrix, in accordance with the theory of thermodynamics [30].

### 3.3. Optimal Optimization Range of the CNT/GN-RTV Preparation Process

The response surfaces of the produced samples were tested for sensing and mechanical qualities at various CNT contents, GN contents, mixing durations, and curing temperatures. The goal of the optimization research for sensing and mechanical characteristics is to simultaneously optimize the sensitivity and the compression modulus of CNT/GN-RTV composites. Additionally, using an expectation function to optimize the many response parameters, while being constrained by maximum expectation restrictions, was a successful strategy for improving multiple response values [31]. The response parameters are provided in Table 5 and optimization ranges for each of the components should be developed. With an important index of 3, the four variables of CNT content, GN content, mixing time, and curing temperature were assumed to be above the median of their respective ranges. The sensitivity and compression modulus response values were chosen to fall within their respective ranges, which were the highest values, with an importance index of 5. These ranges were established by the top limits of the experimental data, which were based on the experimental data.

**Figure 7:**
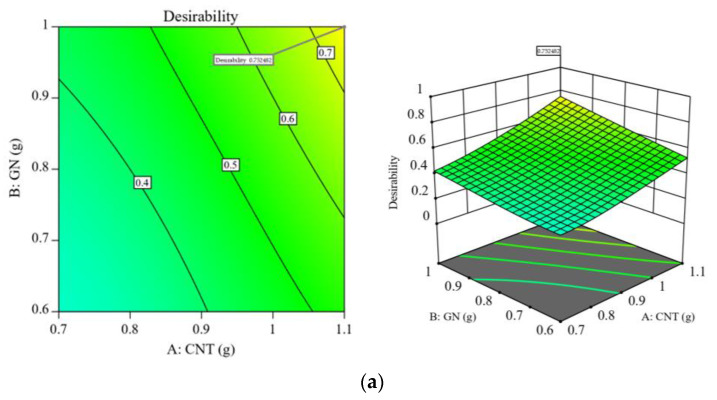

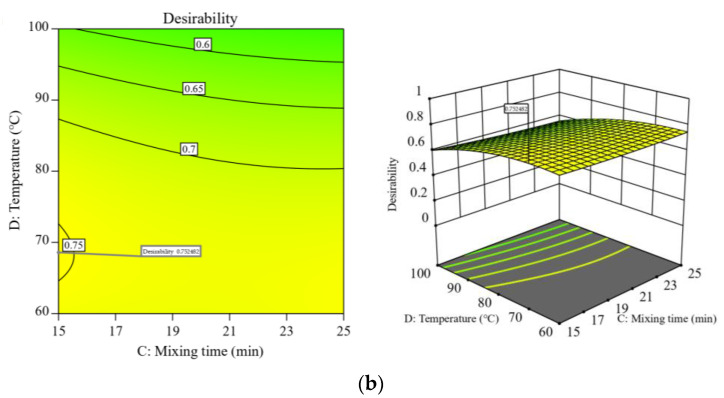
A 3D response surface plot (**a**) with 2D contour plot; (**b**) with a desirability function applied to multiple responses.

**Figure 8:**
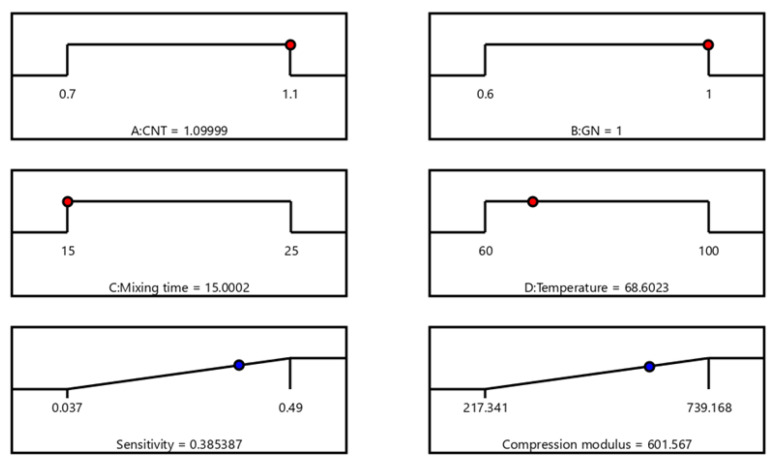
Desirability graph variables for the numerical optimization of four independent variables and two response values.

Figure 7a,b displays the total expectation functions of the sensitivity and compression modulus when they are applied simultaneously to comment responses. The best overall expected value of 75.2% was reached at this point, as shown in Figure 8, when the CNT and GN contents were 1.1 g and 1.0 g, respectively, with a mixing period of 15 min and a curing temperature of 68.6 °C. As shown in Table 5, the taken total starting expectation and response expectation values tended to be close to the optimal conditions. This indicates that the CNT/GN-RTV composites were well-optimized. The improved CNT/GN-RTV composite may, therefore, accomplish a sensitivity of 0.385 kPa^−1^ and a compressive modulus of 601.567 kPa under these circumstances.

## 4. Conclusions

In this study, CNT/GN-RTV nanocomposites were created using mechanical mixing. On the sensitivity and compression modulus, the impacts of four independent variable parameters, namely CNT content, GN content, mixing duration, and curing temperature, were studied. The experimental plan was devised utilizing the central composite design concept, and the response surface model optimization of the sensitivity and compression modulus was developed and evaluated using ANOVA. The developed sensitivity ($R^{2}=0.9634$) and compression modulus ($R^{2}=0.9115$) models also fit rather well, confirming the reliability of the models. Using three-dimensional response surfaces and contour plots, the degree of interactions between CNT content, GN content, the mixing time, and the curing temperature were measured. The experimental design demonstrates that the increase in CNT and GN content has a significant impact on the sensitivity, whereas the high mixing time and curing temperature damage the “3D conductive network” and the interface effect, thereby reducing the sensitivity; however, the mixing time and curing temperature have a substantial impact on the compression modulus. However, the mixing time and curing temperature have a higher impact on the compression modulus, which decreases with increasing temperature and increases sensitivity. On the basis of experimental data and optimization findings, the ideal process parameters for the creation of CNT/GN-RTV nanocomposites were determined to be a CNT content of 1.1 g, a GN content of 1.0 g, a mixing time of 15 min, and a curing temperature of 68.6 °C, respectively. Under these circumstances, the compressive modulus and sensitivity might reach 0.385 kPa^−1^ and 601.567 kPa, respectively. This, indeed, proves the capability of RSM with CCD as an excellent optimization tool for this study and other prospective work, and provides a new idea for the preparation of sensor haptic units.

## Figures and Tables

**Table 1:** Values and levels of chosen variables for central composite design.

Variable	Symbol	Actual Range	Coded Levels				
			−2	Low (−1)	Center (0)	High (+1)	+2
Amount of CNT (g)	A	0.8—1.0	0.7	0.8	0.9	1.0	1.1
Amount of GN (g)	B	0.7—0.9	0.6	0.7	0.8	0.9	1.0
Mixing time (min)	C	15—25	10	15	20	25	30
Temperature (°C)	D	60—100	40	60	80	100	120

**Table 2:** Experimental design matrix and the values of responses based on the experiment run.

	Input Variables (Factors)				Responses	
Run	A: CNT (g)	B: GN (g)	C: Mixing Time (min)	D: Temperature (°C)	Sensitivity (kPa^−1^)	Compression Modulus (kPa)
1	1	1	−1	−1	0.387	559.704
2	0	0	0	0	0.129	636.943
3	0	0	0	0	0.157	651.57
4	0	0	−2	0	0.205	602.178
5	0	−2	0	0	0.197	527.573
6	−1	−1	−1	1	0.07	469.795
7	0	0	0	0	0.13	622.693
8	−2	0	0	0	0.062	579.748
9	1	1	1	1	0.203	557.687
10	0	0	2	0	0.142	687.158
11	1	−1	1	−1	0.192	690.491
12	1	−1	−1	1	0.239	359.624
13	0	0	0	0	0.147	484.934
14	−1	−1	−1	−1	0.09	739.168
15	−1	−1	1	1	0.102	434.897
16	−1	−1	1	−1	0.137	702.085
17	0	0	0	0	0.124	595.274
18	−1	1	−1	1	0.062	394.148
19	0	2	0	0	0.21	580.638
20	0	0	0	2	0.085	217.341
21	−1	1	1	1	0.037	440.39
22	1	1	1	−1	0.322	681.951
23	2	0	0	0	0.49	687.609
24	0	0	0	0	0.166	583.281
25	−1	1	1	−1	0.176	557.612
26	1	1	−1	1	0.338	511.452
27	−1	1	−1	−1	0.19	568.908
28	1	−1	−1	−1	0.22	490.337
29	0	0	0	−2	0.136	589.762
30	1	−1	1	1	0.26	414.272

**Table 3:** Analyses of variance (ANOVA) for response surface model for the sensitivity of CNT/GN-RTV composites using CCD.

Source	Sum of Squares	df	Mean Square	*F*-Value	*p*-Value	Status
Model	0.2862	14	0.0204	28.18	<0.0001	significant
A-CNT	0.1931	1	0.1931	266.26	<0.0001	
B-GN	0.0077	1	0.0077	10.67	0.0052	
C-Mixing Time	0.0036	1	0.0036	4.93	0.0422	
D-Temperature	0.0106	1	0.0106	14.65	0.0016	
AB	0.0047	1	0.0047	6.42	0.0229	
AC	0.0038	1	0.0038	5.26	0.0367	
AD	0.0036	1	0.0036	5.00	0.0409	
BC	0.0060	1	0.0060	8.33	0.0113	
BD	0.0136	1	0.0136	18.79	0.0006	
CD	0.0001	1	0.0001	0.1903	0.6689	
$A^{2}$	0.0302	1	0.0302	41.57	<0.0001	
$B^{2}$	0.0062	1	0.0062	8.54	0.0105	
$C^{2}$	0.0016	1	0.0016	2.14	0.1637	
$D^{2}$	0.0019	1	0.0019	2.55	0.1309	
Residual	0.0109	15	0.0007			
Lack-of-Fit	0.0094	10	0.0009	3.22	0.1045	not significant
Pure Error	0.0015	5	0.0003			
Cor Total	0.2970	29				
$R^{2}$			0.9634			
$R_{Adj}^{2}$			0.9292			
$R_{Pred}^{2}$			0.8103			
CV%			14.95			
Model Precision			22.9879			

**Table 4:** Analyses of variance (ANOVA) for the response surface model for the compression modulus of CNT/GN-RTV composites using CCD.

Source	Sum of Squares	df	Mean Square	*F*-Value	*p*-Value	Status
Model	3.623 × $10^{5}$	14	25,876.54	11.03	<0.0001	significant
A-CNT	1264.94	1	1264.94	0.5394	0.4740	
B-GN	249.05	1	249.05	0.1062	0.7490	
C-Mixing time	12,890.35	1	12,890.35	5.50	0.0332	
D-Temperature	1.931 × $10^{5}$	1	1.931 × $10^{5}$	82.35	<0.0001	
AB	34,313.58	1	34,313.58	14.63	0.0017	
AC	13,243.35	1	13,243.35	5.65	0.0312	
AD	3878.02	1	3878.02	1.65	0.2179	
BC	26.54	1	26.54	0.0113	0.9167	
BD	14,339.76	1	14,339.76	6.12	0.0259	
CD	1636.10	1	1636.10	0.6977	0.4167	
$A^{2}$	861.48	1	861.48	0.3674	0.5535	
$B^{2}$	5600.22	1	5600.22	2.39	0.1431	
$C^{2}$	1913.15	1	1913.15	0.8159	0.3807	
$D^{2}$	73,960.10	1	73,960.10	31.54	<0.0001	
Residual	35,174.54	15	2344.97			
Lack of Fit	17,200.00	10	1720.00	0.4785	0.8497	not significant
Pure Error	17,974.54	5	3594.91			
Cor Total	3.974 × $10^{5}$	29				
$R^{2}$			0.9115			
$R_{Adj}^{2}$			0.8289			
$R_{Pred}^{2}$			0.6856			
CV%			8.74			
Model Precision			14.8684			

**Table 5:** Specification for factors and responses with weightage and importance.

Name	Goal	Lower Limit	Upper Limit	Lower Weight	Upper Weight	Importance	Desirability
A: CNT	in range	0.7	1.1	1	1	3	1
B: GN	in range	0.6	1	1	1	3	1
C: Mixing time	in range	15	25	1	1	3	1
D: Temperature	in range	60	100	1	1	3	1
Sensitivity	maximize	0.037	0.49	1	1	5	0.787
Compression modulus	maximize	217.341	739.168	1	1	5	0.963

## Data Availability

The data presented in this study are available in this article.
